# The Protective Effects of Gadolinum Chloride on Pneumotoxic Effects of Styrene in Rat 

**DOI:** 10.22074/cellj.2015.3

**Published:** 2015-10-07

**Authors:** Mohammad Reza Arab, Ramazan Mirzaei, Fereydoon Sargolzaei Aval

**Affiliations:** 1 Cell and Molecular Research Center, Department of Anatomical Sciences, Faculty of Medicine, Zahedan University of Medical Sciences, Zahedan, Iran; 2 Health Promotion Research Center, Faculty of Health, Zahedan University of Medical Sciences, Zahedan, Iran

**Keywords:** Styrene, Gadolinum, Respiratory, Toxic, Rat

## Abstract

**Objective:**

The aim of the present study was to evaluate the protective effects of gadoli- num on pneumotoxic effects of styrene in rats as an experimental model.

**Materials and Methods:**

In this experimental study a total number of 40 adult male Sprague Dawley rats that weighed 200 ± 13 g were randomly divided into five groups: i. styrene (St, N=10), ii. styrene+gadolinium chloride (GdCl_3_, N=10), iii. control (N=10), iv. GdCl_3_ (N=5) and v. normal saline (Nor.Sal, as a solvent of GdCl_3_, N=5). Normal saline, as a sham control group, was otherwise treated identically. Rats from the experimental groups were exposed to St in an exposure chamber for 6 days/week, 4 hours/day for up to 3 weeks. At the end of the experi- ment, rats from all groups were killed by deep anesthesia. Their lungs were removed, then fixed in formalin and weighed. Tissue samples were processed routinely and sections stained by the hematoxylin and eosin (H&E) and periodic acid Schiff (PAS) methods. We measured the thicknesses of the respiratory epithelia and interalveolar septa. Obtained data were ana- lyzed by ANOVA, the Tukey test and the paired t test.

**Results:**

Shedding of apical cytoplasm in the bronchiole was a prominent feature of the St group. PAS staining revealed histochemical changes in goblet cells in the epithelium of the St group. While there were no significant changes in lung weights and respiratory epithelial thicknesses between all studied groups, statistical analysis showed a significant alteration in the thickness of interalveolar septa in the St and St+GdCl_3_ group compared to the control groups (P<0.001).

**Conclusion:**

Styrene induced structural and histochemical changes in bronchiole, interalveolar septa and alveolar organization in the rats’ lungs. Gadolinium appeared to partially reduce the toxic effects of styrene on the lungs.

## Introduction

Styrene is an organic solvent frequently used in the plastic and resin industries. Although the metabolites of this organic solvent are known carcinogens for humans and animals, a detailed mechanism has not yet been identified. Exposure to this organic solvent usually occurs by breathing, in this way the solvent enters the blood stream and metabolizes in the liver. Working obligations expose a number of people to this organic solvent, however because of the home uses of styrene, all individuals have a chance for exposure to styrene (St) (1). Despite numerous studies to evaluate the ability of styrene metabolism in human lungs, a number of unanswered questions remain. Cruzan et al. (2) have reported that the ability of the human lung to metabolize St is 100 times lower than rats. Another study has indicated that decreasing levels of glutathione (GSH) in lungs can be one of the reasons that lead to an elevated chance for tumorogenesis in laboratory animals (3). The use of St products as preserving containers in food industries is the main reason fordaily encounters to styrene fumes. This can increase the risk of health hazards due to adverse applications of St (4). In addition to the lung effects, researchers have observed many adverse reproductive effects of St and St metabolites. These effects include a decrease in spermatozoid count and a reduction in semen quality in exposed workers (5). 

Although the brain is the main organ to investigate the side effects of St and other relative products, an evaluation of the effects of this solvent on the respiratory tract is necessaryin order to determine its toxic effects in the lungs. These studies have emphasized the importance of the function of the GSH system. Numerous histopathological changes have been reported following the simultaneous administration of St and alcohol in laboratory animals. Coccini et al. (6) showed that co-administration of St and ethanol induced an increase in thickness of the interalveolar septa due to increased synthesis of collagen fibrils. The sensitivity of animals to St varies from species to species, additionally, different organs also respond differently to this organic solvent. There are reports on chronic exposure effects of this pollutant, including decreased capacity of Clara cells to histological staining and hyperplasia in epithelium of terminal bronchioles. Despite efforts to determine the effective concentrations of this solvent to demonstrate these changes inlung, to date, there is a lack of consensus (7). 

There are reports that co-administration of compositions such as gadolinium chloride (GdCl_3_) can reduce the toxic effects of St and its related metabolites. GdCl_3_reduces accumulation of macrophages in tissue by induction of apoptosis however it has little effect on polymorphonuclear inflammatory cells (8). It is also reported that toxic effects of St on the lungs is much higher than its systemic effects (9). Styrene in the lungs metabolizes to St oxide which seems to have many genotoxic effects. Clara cells of the respiratory epithelium are responsible for the metabolism of St. Moreover, the capability of mice cells to metabolize St is higher than that of rats. Use of this solvent in plastic and home appliance industries strongly increases the chance for people to encounter this product (10). Therefore, the lungsare one of the main organs to be examined for the potential toxic effects of this organic solvent in human and animal models. The risks of addiction-like uses of organic solvent cause health problems (11,12). Due to the increasing widespread use of St in our society and the potential toxic effects of this solvent on the pulmonary system, we aim to investigate the protective effects of GdCl_3_on pneumotoxic effects of styrene in rat as an experimental model in an exposure chamber. 

## Materials and Methods

### Animals

**Fig.1:**
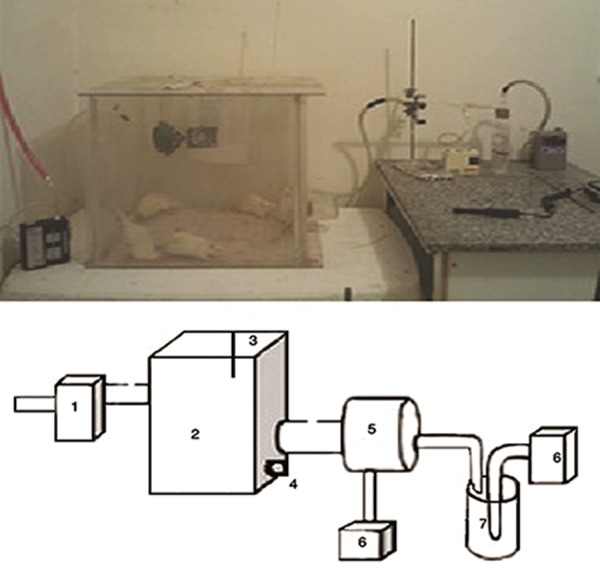
Exposure chamber (top) and schematic diagram for different parts of the exposure system (bottom). 1. Pull pump, 2. Exposure chamber with small fans to homogenize internal air contents of the chamber, 3. Thermometer, 4. Tap water, 5. Mix chamber, 6. Push pump and 7. Impinger.

In this experimental study, we obtained 40 Sprague Dawley rats from Pasteur Institute (Karaj, Iran). The animals were maintained in an animal house at Zahedan University of Medical Sciences under standard living conditions (12 hours day light, 22-24˚C, 45-50% humidity and suitable ventilation) with free access to water and food. These rats weighed 200 ±13 g and were divided randomly into five experimental and control groups: i. St (Merck, Germany) group (N=10), ii. St and GdCl_3_(St+GdCl_3_, Merck, Germany) group (N=10), iii. control group (N=10), iv. GdCl_3_group (N=5) and v. normal saline group (Nor.Sal, N=5) (8). The Nor.Sal group, as the sham control group, was otherwise treated identically. Rats received intraperitoneal injections of GdCl_3_(5 mg/kg) at the beginning of each week. Rats from the experimental groups were exposed to styrene vapor in an exposure chamber for 4 hours per day (7-11 am), 6 days per week for up to 3 weeks as a subacute toxicological study (6). The dimensions of the exposure chamber were 0.5×0.5×0.5 m. To obtain the required exposure concentration of styrene, a push pump forced the air into an impinger which conducted air into the mix chamber, after which it was conducted into the exposure chamber. The internal sealed air content of the exposure chamber was ventilated via a push pump and three small fans were used to homogenate the styrene concentration in the exposure chamber (Fig .1). The exposure conditions were fixed and the St level of the chamber was measured hourly by standard methods (Phocheck 5000, Ion Science, England). The ventilation rate of the exposure chamber was adjusted at 1.75 per hour. Air capacity of the chamber was 0.125 m_3_and its temperature was fixed at 24˚C during all procedures. The concentration The Protective Effects of Gadolinum of St vapor in the exposure chamber was fixed at 1320 ± 140 part per million (ppm) during the experiment (Fig .1). All study rats were weighed before and after the examinations. According to the schedule, at the end of the examination period, all rats in the control and experimental groups were subjected initially to a deep anesthesia with chloroform prior to sacrifice, followed by removal of their lungs. All animal procedures were approved by the Ethics Committee at Zahedan University of Medical Sciences (90-332/2011). 

### Histological examinations

According to routine histological methods, lung samples were processed in 10% formalin (Merck, Germany). Paraffin blocks were prepared by cutting into sections of 6 µm in thickness by a rotary microtome (Leica, Germany), after which they were stained with hematoxylin and eosin (H&E, Merck, Germany) (13). To measure the thickness of respiratory epithelia and interalveolar septa, a Leica DM500 microscope was calibrated with a Zeiss stage micrometer equipped with special software. Data were recorded with a ×40 objective lens. From each slide, we studied 5-10 areas and measured at least 50 bronchioles and interalveolar septa. 

### Statistical analysis

Data were analyzed by SPSS software using the paired t test and ANOVA. A P value of <0.05 was considered statistically significant. 

## Results

**Fig.2:**
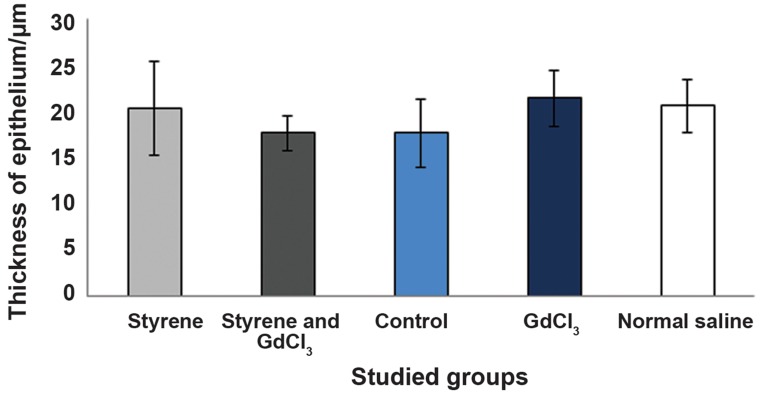
Comparison of thickness of respiratory epithelium in different groups. Gdcl_3_; Gadolinium chloride.

Prior to the experiment, the rats weighed 188 ± 9.32 g (control), 195 ± 14 g (St), 223 ± 27.1 g (St+GdCl_3_), 205 ± 9.9 g (GdCl_3_) and 206 ± 9.9 g (Nor.Sal). We observed no significant differences in rats’ weights in the control and experimental groups before and after chemical exposures. There was no significant difference in lung and trachea weights between control and experimental groups. The Tukey test for measurements of respiratory epithelial thicknesses also showed no significant difference between experimental and control groups (Fig .2). However, there was a significant difference for the thickness of interalveolar septa among all studied groups, with the exception of the Nor.Sal group when compared to the control group (P<0.001, Table 1). 

**Table 1:** Comparison of measured variables in rats after administration of styrene and gadolinium chloride (GdCl_3_ in exposure chamber)


Groups	Control (untreated)	Styrene (St)	St+GdCl_3_	Normal saline GdCl_3_	(Nor.Sal)
Variables

Lung weight (g)	1.89 ± 0.22	1.71 ± 0.26	1.95 ± 0.19	1.7 ± 0.14	2.1 ± 0.17
Respiratory epithelium (µm)	17.7 ± 3.65	20.34 ± 5.07	17.7 ± 1.89	21.48 ± 3.02	20.66 ± 2.86
ANOVA	ANOVA -not significant, P>0.05				
P value*					
Interalveolar septa (µm)	5.5 ± 1.37	13.14 ± 1.5	20.37 ± 3.31	9.29 ± 3.54	6.3 ± 2.22
ANOVA	ANOVA P<0.001				
P value**										


*; ANOVA did not show any significant difference for respiratory epithelium between experimental and control groups, **; Tukey test showed significant difference for interalveolar septa between St and St+GdCl_3_ with control groups (P<0.001) and Gdcl_3_ ; Gadolinium chloride.

**Fig.3:**
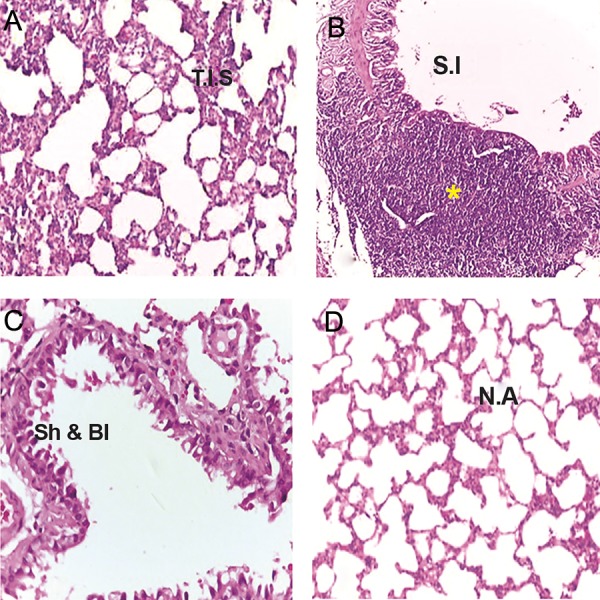
Thickened interalveolar septa (T.I.S). A. Severe inflammation (S.I), B. Shedding and blebbing (Sh and Bl) accompanied with necrosis, C. Large bronchiole in the styrene (St) group and D. Normal alveoli (N.A) in the control group. Hematoxylin and eosin (H&E), ×100 (A, D), ×400 (B, C). *; Aggregation of lymphoid cells.

The control group had evidence of normal respiratory epithelial architecture with some goblet cells in the bronchioles and normal thicknesses for interalveolar septa and alveoli. An increase in the thicknesses of interalveolar septa associated with interstitial inflammation of lung tissue, along with an increase in inflammatory cells was evident in the St group. Histological examination demonstrated that the St+GdCl_3_ group had less severe reactions than the St group. Accordingly, there were a reduced number of inflammatory cells (macrophages) in the walls of large and small bronchioles in the St+GdCl_3_ group. Shedding of apical cytoplasm within the epithelium of bronchioles and formation of blebs in the respiratory epithelia was evident in the St group (Fig .3 A-D).

## Discussion

The current study results indicated an increase in the thickness of interalveolar septa with interstitial inflammation, an alteration in the respiratory epithelia of bronchioles along with a decrease in their thicknesses due to the loss of apical cytoplasm in the experimental compared to the control groups. 

Despite a non-significant difference in rats’ weights before and after exposure to St, weights of the trachea and lungs, and thickness of respiratory epithelia in the bronchioles, statistical analysis revealed significant changes in the thicknesses of the interalveolar septa between the experimental and control groups. 

Exposure to St vapor in working areas is usually through respiratory routes. Studies have shown that 60-70% of St is absorbed in the respiratory epithelium. In these cells, styrene turns into intermediate metabolites which are finally excreted with the urine in the form of mandelic and phenyl glyoxylic acids. Pulmonary complications such as congestion, hemorrhage, edema and infiltration of inflammatory cells have been reported in a review article (14). The obtained data in our study agreed with these findings. 

In humans, absorbed styrene turns into hippuric acid, however it should be emphasized that this capability in humansis very low. Studies have shown that confounding factors such as smoking, a history of lung disease, gender and age could intensify the harmful induced effects of styrene in tissues. 

Among workers poisoned with styrene, neurotoxic effects of styrene might lead to serious health risks (3,12). 

Worldwide, epidemiological studies have indicated a need for precise data collection to determine acute and chronic effects of working place pollutants (15). Exposure to St vapor (1000 ppm) for one year resulted in increased weight of the liver and decreased whole body weight. The aforementioned report indicated that responses of different tissues to St differed and showed species specific patterns (2). 

In the current study, we observed no significant difference between the weight of rats before and after the experiment, which seemed to be related to the duration of the experiments. Changes in the thicknesses of interalveolar septa were also reported by Coccini et al. (16) following exposure to styrene. Cruzan et al. (2) also reported predictable hyperplasia in the bronchioles of rats following exposure to St. Cellular loss and necrosis, loss of apical cytoplasm and drop shape epithelial cells in the respiratory epithelium were qualitative results of the current study.

We have chosen the bronchioles, where the majority of styrene is absorbed and metabolized, as the target to measure the thickness of respiratory epithelium. We also observed a decline in the staining intensity of respiratory epithelial cells and their accumulation, which was also reported by Cruzan et al. (2). Our findings demonstrated significant changes in the thickness of interalveolar septa after exposure to St in rats which was compatible with the results of Coccini et al. (6). Lipophilic metabolites of styrene such as St oxide were the main mutagenic factors that induced cancer in the laboratory animals. Altered staining properties of Clara cells were one of the most important findings of the current study. Kaufman et al. (17) reported an increase in cell proliferation in lung alveoli which could explain the observed increase in the thickness of interalveolar septa observed in our study. 

Despitethe high health risks of styrene and its metabolites in plastic and resin workers, there is still little consensus regarding its mechanism of action and relation with lung cancer (17). By induction of apoptosis in macrophages, GdCl_3_prevents their accumulation in the tissues and therefore prohibits the release of more cytokines, elevation of inflammatory reactions, and tissue destruction (18,19). It is accepted that numerous chemical agents in our daily lives or working environment have genotoxic effects (13). GdCl_3_reduces free radical production in lung tissue and by this mechanism can prevent the toxic effects to tissues which occur following tissue ischemia reperfusion (19,20). Mogel et al. (21), have shown that after exposure to aromatic compounds such as St, the expression level of cyclooxygenase-2 (COX-2) increased which in turn upregulated prostaglandins (PGs) PGE _2_and PGF_2α_as the major products of COX2. The level of pollutants in indoor environments increased compared with outdoor environments because of widespread use of volatile organic compounds. Study showed that these compounds caused airway inflammation and alterations in the immune system (21). Tissue damage due to inhalation of St and other aromatic compounds might be related to oxidative stress. Styrene tissue injuries might be attributed to decreases in the GSH system as a main antioxidant system (22). GdCl_3_has been shown to inhibit macrophage migration and activation *in vivo* therefore preventing lung injury induced by ozone inhalation (23). This property of GdCl_3_might be the reason why we observed less macrophages than expected. 

## Conclusion

Tissue responses to styrene and probably to its metabolites pass from a pattern of inflammation, proliferation and reduction of staining properties of epithelial cells with loss of apical cytoplasm and a decreasein the number of goblet cells. GdCl_3_appears to decrease the severity of reactions to styrene by modulating through the activity of inflammatory cells. It is suggested that the extent of fibrous portion extra cellular matrix (ECM) have been changed. 
